# Validation of Recorded Diagnoses of Acute Kidney Injury Among Surgical Patients in the Japanese Diagnosis Procedure Combination Database

**DOI:** 10.2188/jea.JE20250387

**Published:** 2026-06-05

**Authors:** So Sato, Akira Okada, Yoshihisa Miyamoto, Hayato Yamana, Hideo Yasunaga

**Affiliations:** 1 Department of Clinical Epidemiology and Health Economics, Graduate School of Medicine, The University of Tokyo, Tokyo, Japan; 2 Department of Prevention of Diabetes and Lifestyle-Related Diseases, Graduate School of Medicine, The University of Tokyo, Tokyo, Japan; 3 Department of Real-World Evidence, Graduate School of Medicine, The University of Tokyo, Tokyo, Japan; 4 Data Science Center, Jichi Medical University, Tochigi, Japan

**Keywords:** acute kidney injury, validation, diagnosis, laboratory data, administrative data

## Abstract

**Background:**

Researchers widely use the Diagnosis Procedure Combination (DPC) database for studies on acute-care hospitalization in Japan. However, the validity of acute kidney injury (AKI) diagnostic codes among patients who underwent surgery under general anesthesia is unclear.

**Methods:**

Under the Japanese Next-Generation Healthcare Infrastructure Act, we obtained DPC data linked with the laboratory data of adult surgical patients with baseline and follow-up serum creatinine measurements from three hospitals (2021–2024). Recorded diagnoses of AKI were identified using the International Classification of Diseases–10^th^ Revision codes (N17.x). The reference standard was the diagnosis of AKI per the creatinine-based Kidney Disease: Improving Global Outcomes (KDIGO) criteria. The sensitivity, specificity, likelihood ratios, and predictive values were calculated. We evaluated the coding validity for identifying AKI stages 2 and 3. Subgroup analyses were conducted according to baseline estimated glomerular filtration rates (eGFR) (≥60.0, 30.0–59.9, 15.0–29.9 mL/min/1.73 m^2^), type of admission (planned/emergent), and surgery type (cardiovascular/non-cardiovascular).

**Results:**

Among 7,374 eligible patients, 663 (9.0%) met the KDIGO-defined AKI criteria, while 35 (0.5%) patients had the AKI codes. The sensitivity was 4.1% (95% CI, 2.7–5.9%), and the specificity was 99.9% (95% CI, 99.8–99.9%). The sensitivity increased for patients with stages 2 and 3 AKI, lower eGFR, emergent admission, or cardiovascular surgery.

**Conclusion:**

Among surgical patients, AKI coding in the DPC exhibited high specificity and positive predictive value, albeit low sensitivity. Incorporating laboratory data is important for more accurate identification of patients with AKI for use in clinical studies.

## INTRODUCTION

The use of routinely collected healthcare data in clinical studies is expanding globally.^1^^,^^2^ Although such data offer valuable opportunities for clinical and epidemiological research, their utility is contingent upon the accuracy of the information they contain.^3^ Inaccurate records of diagnoses can lead to misclassification of exposures and outcomes, thereby introducing bias into study findings. To avert such erroneous classification, researchers must evaluate the accuracy with which key diagnoses are captured.

Acute kidney injury (AKI) is a common complication in various populations that is associated with poor outcomes, including higher mortality or subsequent chronic kidney disease (CKD) progression.^4^^,^^5^ Surgery is one of the important contributors to AKI,^6^ particularly cardiovascular surgery.^7^ Previous studies have shown that surgical patients who developed AKI experienced significantly higher mortality compared with those without AKI.^8^^,^^9^ Therefore, validating the accuracy of the recorded diagnoses of AKI in healthcare databases is essential to ensure reliable research in this field. The surgical context also offers the following practical advantages for validation studies: 1) preoperative testing protocols often ensure that baseline creatinine values are measured within the same institution; 2) the timing when the kidneys are exposed to injury, mainly due to surgical stress, is usually well-defined; and 3) patients are routinely monitored, including serial measurements of serum creatinine.

Several validation studies have assessed the records of AKI coded with the International Classification of Diseases (ICD).^10^^–^^17^ These studies consistently reported low sensitivity and high specificity of AKI coding against the reference standard, based on creatinine measurements, including the Kidney Disease: Improving Global Outcomes (KDIGO) criteria.^18^ The sensitivity generally increased when the AKI definition was limited to more severe AKI, and the validity of coding tended to be higher among patients receiving renal replacement therapy.^10^^–^^15^ Additionally, some studies observed a trend toward improved validity of AKI coding in more recent years.^10^^–^^13^

Despite the widespread adoption of the KDIGO criteria since their publication in 2012,^18^ validation studies for AKI coding specifically targeting surgical populations remain limited. Because coding accuracy can vary over time and across healthcare systems or countries,^10^^–^^13^ evaluating the validity of coding of AKI is essential in Japan, where the ICD–10^th^ Revision (ICD-10) is currently used. Moreover, while prior validation studies suggest that coding performance can vary across clinical conditions, such as the severity of CKD,^10^ its validity in patients undergoing surgery under general anesthesia has not been thoroughly established.

This study aimed to assess the validity of ICD-10 coding of AKI in patients who underwent surgery under general anesthesia, using the Japanese Diagnosis Procedure Combination (DPC) data. We also examined whether the diagnostic performance varied by the AKI stage used as the reference standard, as well as by subgroups, such as CKD severity, type of admission, and type of surgery.

## METHODS

### Data

This retrospective validation study analyzed administrative claims data and discharge abstracts from the DPC system,^19^ along with laboratory data recorded through the Standardized Structured Medical Record Information Exchange (SS-MIX).^20^

The DPC contains unique hospital and patient identifiers (each hospitalization was assigned a unique identifier), patient age at admission, sex, dates of admission and discharge, and diagnoses. The diagnostic data covers the primary condition, comorbidities at admission, and complications during hospitalization, recorded using both Japanese text and ICD-10 codes. The database also includes interventional and surgical procedures recorded using unique Japanese procedural codes. Attending physicians document all clinical details at discharge. Outpatient records are also part of the DPC database and include unique identifiers, medical procedures, and corresponding dates. Approximately 1,800 acute care hospitals have adopted the DPC system.

The SS-MIX system standardizes medical chart data, including daily laboratory test results from various vendors. Over 1,200 medical institutes have implemented the SS-MIX system. Laboratory tests are categorized using Japanese Laboratory Code Version 10 (JLAC-10) codes.

Data were obtained from each medical institution by certified processors from the General Incorporated Foundation for Fair and Safe Use of Anonymized Standardized Health Data of Japan. All records were anonymized and linked at the individual level under the Japanese Next-Generation Healthcare Infrastructure Act.^21^ Three acute-care hospitals contributed data spanning January 2019 to December 2024, including one large hospital with approximately 1,300 beds in the Kyushu region, one medium-sized hospital with approximately 300 beds also in Kyushu, and one medium-sized hospital with approximately 300 beds in the Kanto region. These institutions were selected based on their implementation of both the SS-MIX and DPC systems, along with the existing data-sharing agreements with the Foundation. The periods of data availability and patient inclusion period differed across institutions and between the DPC and SS-MIX data; additional information is available in eTable 1.

This study was conducted under the framework of the Japanese Next-Generation Healthcare Infrastructure Act,^21^ exempting it from requiring approval by an independent ethics committee. The study was registered with the Ethics Committee of the University of Tokyo (registration number 2025161NIe). The need for written informed consent was waived due to the use of anonymized data. The manuscript adheres to the Standards for Reporting of Diagnostic Accuracy Studies 2015.^22^

### Sample

We enrolled patients aged ≥18 years who underwent surgery under general anesthesia, had undergone at least one serum creatinine measurement 7–365 days before the surgery, and had undergone at least one follow-up serum creatinine measurement within 2 days after surgery. The exclusion criteria were as follows: patients who did not undergo surgery within 7 days after hospital admission; those who received a renal transplant (ICD-10 code Z94.0, procedure codes: K780 or K780-2); those who underwent hemodialysis (procedure code: J038) or peritoneal dialysis (procedure code: J042) under a diagnosis of end-stage renal disease documented as a comorbidity at admission, or those who received dialysis before or on the day of surgery; and patients with a baseline serum creatinine level ≥4 mg/dL or an estimated glomerular filtration rate (eGFR) <15 mL/min/1.73 m^2^. If a patient had undergone multiple surgeries during a single hospital stay, we focused only on the first surgery. Multiple hospitalizations for the same patient were counted as separate cases because each hospitalization was assigned a unique identifier in our dataset, even for the same individual.

### Outcomes

The primary outcome of this study was the AKI coding based on ICD-10 codes N17.x recorded as complications during hospitalization in the DPC data.^11^^,^^12^^,^^15^^,^^17^ The reference standard was the diagnosis of AKI based on the KDIGO criteria (eTable 2).^18^ Specifically, AKI was identified as either a ≥1.5-fold increase in serum creatinine from baseline or an absolute increase of ≥0.3 mg/dL within 2 days. AKI severity was categorized into three stages: stage 1 included an absolute increase of ≥0.3 mg/dL or a 1.5–1.9-fold increase from baseline; stage 2 corresponded to a 2.0–2.9-fold increase; and stage 3 was defined as a ≥3.0-fold increase, a serum creatinine level of ≥4 mg/dL, or the initiation of renal replacement therapy. When multiple test results were recorded on the same day during hospitalization, the highest value was used for the assessment of AKI status. We extracted all serum creatinine test results from 365 days before surgery through the date of hospital discharge. The baseline value was calculated as the mean serum creatinine measured in outpatient settings during 7–365 days before surgery, based on the approach adopted by a previous study^23^ (eFigure 1). The baseline eGFR for men was calculated using the following formula for Japanese individuals: eGFR = 194 × serum creatinine(−1.094) × age(−0.287); the result was multiplied by 0.739 to obtain the eGFR for women.^24^ We defined AKI solely based on changes in serum creatinine because urinary output data were not available in the dataset.

### Other variables

In addition to baseline serum creatinine and baseline eGFR, we collected data on age, sex, Charlson Comorbidity Index,^25^ type of admission (planned or emergent), intensive care unit admission after surgery, type of surgery, baseline serum creatinine, baseline eGFR, and in-hospital death as patient characteristics. Surgery type was organized into 11 categories based on the Japanese medical fee schedule: skin and subcutaneous tissue; musculoskeletal system, limbs, and trunk; nervous system and skull; ophthalmic; otolaryngology; face, mouth, neck; thoracic; cardiovascular; abdominal; urinary tract and adrenal glands; and genital procedures.

### Statistical analysis

We first summarized the background characteristics of the study population. Next, using laboratory-based definitions from the KDIGO criteria as the reference standard, we evaluated the validity of ICD-10 coding of AKI in the DPC data. The validity of ICD-10 coding of AKI was evaluated using metrics, such as sensitivity, specificity, positive predictive value (PPV), negative predictive value (NPV), positive likelihood ratio (LR+), negative likelihood ratio (LR−), and diagnostic odds ratio (DOR). An LR+ of at least 10 was interpreted as strong evidence to consider the possibility of a disease, while an LR− of 0.1 or lower was considered strong evidence to rule out this possibility.^26^

We also examined whether the coding validity differed when using different AKI stages as the reference standard, based on a previous study.^12^ To this end, we assessed the validity of ICD-10 coding against KDIGO stage 2 and stage 3 AKI by restricting the analysis to patients with severe AKI.

Thereafter, we performed subgroup analyses to explore whether the validity of ICD-10 coding of AKI varied by clinical condition. These analyses were exploratory in nature. Subgroups were defined on the basis of baseline eGFR (≥60.0, 30.0–59.9, and 15.0–29.9 mL/min/1.73 m^2^), type of admission (planned or emergency), and surgery type (cardiovascular or non-cardiovascular). Surgery was classified into cardiovascular or non-cardiovascular based on previous findings that identified cardiovascular procedures as having a higher attendant AKI risk.^7^^,^^9^

All analyses were conducted using Stata MP version 19.0 (StataCorp, College Station, TX, USA).

### Sensitivity analysis

We conducted four sensitivity analyses. First, because the KDIGO criteria define AKI as a ≥1.5-fold increase in serum creatinine within 7 days,^18^ we restricted AKI assessment to creatinine changes occurring within 7 days after surgery (sensitivity analysis 1). In sensitivity analysis 2, we used the most recent outpatient creatinine value from 7–365 days before surgery instead of the average from that period. In sensitivity analysis 3, we used the most recent inpatient creatinine value before surgery. A previous study has shown that the results can differ based on the nature of the definition of baseline creatinine,^27^ prompting two additional analyses. The definitions applied in each analysis are illustrated in eFigure 1.

Additionally, to address the possibility that AKI was coded as unspecified renal failure or postprocedural renal failure, we expanded the definition of AKI to include ICD-10 N17.x, N19 (unspecified renal failure), and N99.0 (postprocedural renal failure) recorded as in-hospital complications in the DPC data (sensitivity analysis 4).

## RESULTS

### Patient characteristics

**Figure 1.**
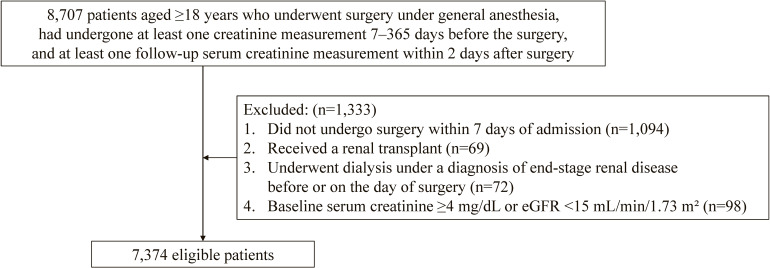
Flowchart of the selection of study patients. eGFR, estimated glomerular filtration rate.

Between September 2021 and December 2024, we identified 8,707 patients who received surgery under general anesthesia and had undergone both baseline and follow-up serum creatinine measurements. After applying the exclusion criteria, 7,374 patients were included in the analysis (Figure 1). A total of 21,410 follow-up serum creatinine tests were performed during hospitalization after surgery, with a mean of 6.3 (standard deviation [SD], 7.0) tests per patient. Table 1 presents the patients’ baseline characteristics. The patients’ mean age was 61.8 (SD, 15.9) years, and 3,453 (46.8%) patients were men. The baseline eGFR was ≥60.0 mL/min/1.73 m^2^ in 5,433 (73.7%) patients, 30–59.9 mL/min/1.73 m^2^ in 1,804 (24.5%) patients, and 15–29.9 mL/min/1.73 m^2^ in 137 (1.9%) patients. Cardiovascular surgeries were performed in 710 (9.6%) patients. Overall, 926 (12.6%) patients were admitted to the intensive care unit after surgery. Emergency admissions accounted for 107 (1.5%) cases. A total of 9 (0.1%) patients died during hospitalization.

**Table 1.** Patient characteristics

Characteristics	Patients who underwent surgery under general anesthesia *N* = 7,374	
Age, years, mean (SD)	61.8	(15.9)
Men, *n* (%)	3,453	(46.8)
Charlson Comorbidity Index, mean (SD)	2.1	(2.1)
Planned hospitalization, *n* (%)	7,267	(98.6)
Type of surgery, *n* (%)		
Skin and subcutaneous tissue	132	(1.8)
Musculoskeletal system, limbs and trunk	1,360	(18.4)
Nervous system and skull	248	(3.4)
Ophthalmic	32	(0.4)
Otolaryngology	186	(2.5)
Face, mouth, neck	238	(3.2)
Thoracic	1,009	(13.9)
Cardiovascular	710	(9.6)
Abdominal	1,736	(23.5)
Urinary tract and adrenal glands	663	(9.0)
Genital	1,060	(14.4)
Intensive care unit admission after surgery, *n* (%)	926	(12.6)
Baseline serum creatinine, mg/dL, mean (SD)	0.8	(0.3)
Baseline eGFR, mL/min/1.73 m^2^, *n* (%)		
≥60.0	5,433	(73.7)
30.0–59.9	1,804	(24.5)
15.0–29.9	137	(1.9)
In-hospital death, *n* (%)	9	(0.1)

eGFR, estimated glomerular filtration rate; SD, standard deviation.

### Validity of ICD-10 coding of AKI for identifying the laboratory diagnosis of AKI

Among the 7,374 eligible patients, 663 (9.0%) met the KDIGO criteria for AKI based on serum creatinine changes, including 48 who received renal replacement therapy after surgery. Of those with KDIGO-based AKI, 504 (6.8%) had stage 1, 82 (1.1%) had stage 2, and 77 (1.0%) had stage 3 AKI. Notably, severe AKI was more common among patients admitted emergently, with stage 1, 2, and 3 observed in 12.2%, 6.5%, and 12.2%, respectively, compared to 6.8%, 1.0%, and 0.9% among those with planned admissions. In contrast, AKI was coded as ICD-10 N17.x representing complications during hospitalization in the DPC data for 35 (0.5%) patients. Table 2 summarizes the AKI coding performance of the DPC data. The overall sensitivity was 4.1% (95% confidence interval [CI], 2.7–5.9%) and the specificity was 99.9% (95% CI, 99.8–99.9%). The PPV was 77.1% (95% CI, 59.9–89.6%), the NPV was 91.3% (95% CI, 90.7–92.0%), the LR+ was 34.2 (95% CI, 15.6–74.9), the LR− was 0.96 (95% CI, 0.95–0.98), and the DOR was 35.6 (95% CI, 16.4–77.1). The sensitivity and LR+ increased when stages 2 and 3 AKI were set as the reference.

**Table 2.** Validity of DPC data for identifying AKI against serum creatinine definitions using the KDIGO criteria among patients who underwent surgery

Type of analysis, subgroup	Frequency (KDIGO-defined criteria)		Frequency (ICD-10 coding)		Sensitivity (95% CI) (%)	Specificity (95% CI) (%)	PPV (95% CI) (%)	NPV (95% CI) (%)	LR+ (95% CI)	LR− (95% CI)	DOR (95% CI)

	*n*	(%)	*n*	(%)							
**Overall**	663	9.0	35	0.5	4.1 (2.7–5.9)	99.9 (99.8–99.9)	77.1 (59.9–89.6)	91.3 (90.7–92.0)	34.2 (15.6–74.9)	0.96 (0.95–0.98)	35.6 (16.4–77.1)
Stages 2 and 3	159	2.2	35	0.5	13.2 (8.4–19.5)	99.8 (99.7–99.9)	60.0 (42.1–76.1)	98.1 (97.8–98.4)	68.1 (35.3–131)	0.87 (0.82–0.92)	78.3 (39.4–155)
**Subgroup analyses**											
**eGFR category, mL/min/1.73 m^2^**											
≥60.0	382	7.0	7	0.1	1.3 (0.4–3.0)	>99.9 (99.9–>99.9)	71.4 (29.0–96.3)	93.1 (92.3–93.7)	33.1 (6.4–170)	0.99 (0.98–>0.99)	33.5 (7.5–^a^)
30.0–59.9	245	13.6	23	1.3	6.9 (4.1–10.9)	99.6 (99.2–99.9)	73.9 (51.6–89.8)	87.2 (85.6–88.7)	18.0 (7.2–45.3)	0.93 (0.90–0.97)	19.3 (7.8–48.0)
15.0–29.9	36	26.3	5	3.6	13.9 (4.7–29.5)	>99.9 (96.4–>99.9)	100 (47.8–100)	76.5 (68.4–83.5)	^a^	0.86 (0.76–0.98)	^a^
**Type of admission**											
Planned	630	8.7	30	0.4	3.5 (2.2–5.2)	99.9 (99.8–99.9)	73.3 (54.1–87.7)	91.6 (90.9–92.2)	29.0 (13.0–64.8)	0.97 (0.95–0.98)	30.0 (13.6–66.3)
Emergent	33	30.8	5	4.7	15.2 (5.1–31.9)	>99.9 (95.1–>99.9)	100 (47.8–100)	72.5 (62.8–80.9)	^a^	0.85 (0.74–0.98)	^a^
**Type of surgery**											
Cardiovascular	172	24.2	31	4.4	14.0 (9.2–20.0)	98.7 (97.3–99.5)	77.4 (58.9–90.4)	78.2 (74.9–81.3)	10.7 (4.7–24.5)	0.87 (0.82–0.93)	12.3 (5.3–28.4)
Non-cardiovascular	491	7.4	4	0.1	0.6 (0.1–1.8)	>99.9 (99.9–>99.9)	75.0 (19.4–99.4)	92.7 (92.0–93.3)	37.7 (3.9–362)	0.99 (0.99–>0.99)	37.9 (5.4–^a^)

AKI, acute kidney injury; CI, confidence interval; DOR, diagnostic odds ratio; eGFR, estimated glomerular filtration rate; ICD-10, International Classification of Diseases, 10^th^ Revision; KDIGO, Kidney Disease: Improving Global Outcomes; LR+, positive likelihood ratio; LR−, negative likelihood ratio; NPV, negative predictive value; and PPV, positive predictive value.

^a^Results not shown due to infinite estimates.

### Subgroup analyses of clinical conditions

AKI coding performance varied by the clinical subgroup. The sensitivity increased with a decline in baseline eGFR, reaching its peak in patients with eGFR 15.0–29.9 mL/min/1.73 m^2^ (13.9%; 95% CI, 4.7–29.5%). In contrast, low sensitivity was observed in patients with preserved kidney function (eGFR ≥60.0) (1.3%; 95% CI, 0.4–3.0%), although the specificity was high (>99.9%; 95% CI, 99.9–>99.9). Emergency admissions also demonstrated higher sensitivity and PPV compared with planned admissions. Among surgery types, cardiovascular procedures had higher AKI prevalence and coding sensitivity compared with non-cardiovascular surgeries. The specificity remained consistently high across all subgroups.

Overall, diagnostic performance was better in patients with higher CKD severity, during emergency admissions, and in patients undergoing cardiovascular surgery.

### Sensitivity analysis

The results across the four sensitivity analyses remained consistent with the main findings (eTables 3, eTable 4, eTable 5 and eTable 6). Sensitivity and PPV showed minimal fluctuation, whereas specificity remained high across all subgroup comparisons.

## DISCUSSION

This study evaluated the validity of ICD-10 coding of AKI using the DPC data in patients who underwent surgery under general anesthesia. Although the overall sensitivity of AKI diagnosis based on ICD-10 coding was low (4.1%), the specificity was very high (99.9%), and the LR+ exceeded 10. These results suggest that, while many true AKI cases may be missed when relying on ICD-10 coding alone, those identified through coding are highly likely to represent true AKI. The sensitivity improved with the rise in AKI severity when higher stages were used as the reference standard. Using ICD-10 coding of AKI alone may not be sufficient for identifying all AKI cases, and supplementation with laboratory data is critical for more accurate assessment.

This study found that, except for sensitivity, the validity of ICD-10 coding for AKI was comparable to that reported by previous validation studies conducted in Japan and other countries (eTable 7).^11^^–^^17^ However, the sensitivity observed in this study was lower than that reported in most previous studies. Although the systematic review did not reveal any clear factors associated with higher sensitivity, three possible explanations may account for this difference. First, in Japan, under the DPC payment system based on the main diagnosis,^19^ there are no financial incentives for attending clinicians to document AKI as a complication during hospitalization. In other countries, including the United Kingdom, the United States, and Canada, ICD-10 coding is used for healthcare reimbursement and resource allocation, which likely increases the drive to ensure accurate coding practices.^12^^,^^28^ Second, because the DPC data are derived from discharge summaries, attending physicians may fail to code AKI that resolved before discharge. A previous study investigating major abdominal surgeries in Japan showed that 84% of patients with laboratory-defined AKI recovered kidney function by discharge.^29^ Third, as in many healthcare systems, AKI in Japan is often diagnosed by non-nephrologists.^30^ This may result in under-recognition or inconsistent documentation, particularly for less severe cases. A study from the United Kingdom reported that only 22–31% patients with AKI consulted with a nephrologist.^31^ Similarly, a study from China found that many non-nephrology departments failed to identify AKI, including 94.5% of cases in orthopedic wards.^32^ In contrast, the sensitivity and PPV of ICD-10 coding of AKI in our study were higher than those reported by a previous AKI coding validation study conducted in Japan.^15^ This may be attributable to the following. First, our study focused on a surgical population that mainly comprised patients undergoing elective surgeries, which allowed for the definition of a reliable baseline creatinine value measured within the same institution. Second, the method used to define baseline serum creatinine may have influenced the results. The previous study relied on any two creatinine values measured within 7 days of each other,^15^ irrespective of whether they were obtained in inpatient or outpatient settings. In contrast, our study defined baseline creatinine as the mean value from outpatient tests conducted within 7–365 days before surgery,^23^ a method that more accurately reflects expert judegment.^27^

The validity of ICD-10 coding of AKI improved when higher stages were used as the reference standard and in specific clinical contexts, consistent with previous studies.^11^^,^^12^^,^^14^ The sensitivity for AKI of greater severity was higher than that of AKI of lesser severity, possibly because clinicians are more likely to detect and document severe cases.^12^^,^^17^ The sensitivity and PPV increased in patients with more advanced stages of CKD. This trend probably reflects greater clinical recognition and documentation of AKI when kidney dysfunction is more apparent, consistent with the findings of studies conducted in other countries.^11^^,^^14^ Emergent admissions also showed better validity of ICD-10 coding of AKI than planned admission, likely due to a higher prevalence of severe AKI. Similarly, the validity of ICD-10 coding of AKI was higher for cardiovascular surgery cases compared with non-cardiovascular cases. These findings may support the cautious use of AKI coding data in scenarios where the diagnostic performance appears relatively stronger.

The high PPV and LR+ support the use of ICD-10 coding for AKI in the DPC database in contexts such as descriptive studies on patient prognosis, as cases identified through these codes are highly likely to represent true AKI. However, the consistently low sensitivity remains a key limitation, indicating that the database is less effective for surveillance or for identifying all AKI cases across the patient population. As demonstrated in this study, when laboratory data are available, researchers should incorporate them to enhance the sensitivity of AKI detection in future studies.

The findings of the study should be interpreted in the context of some limitations. First, the findings may not be applicable to other clinical milieus, such as non-surgical settings. Moreover, because this study predominantly included patients undergoing scheduled elective surgery, the findings may not be generalizable to other patient populations. Further validation studies in broader clinical contexts are needed to overcome these limitations. Second, we used only serum creatinine as the reference standard without incorporating urinary output data to define AKI. This approach may have underestimated the true number of AKI cases as defined by the KDIGO criteria,^18^ although reliable urinary output data are usually difficult to collect, and several studies have considered creatinine measurements as the sole criteria.^12^^–^^17^ Third, because the complications included in the DPC data do not include the timing of diagnosis, the AKI coding might not reflect AKI occurring within 7 days after surgery but may rather represent AKI episodes that could not be directly attributed to the surgery. Fourth, although the study included a large sample, data were derived from only three medical institutions that had implemented the SS-MIX system and provided data under the Next-Generation Healthcare Infrastructure Act. Therefore, the findings may not be generalizable to smaller institutions or those without similar data infrastructure. Lastly, because different patient identifiers were assigned for each admission in our data, we could not examine whether coding decisions during one hospitalization affected those of later admissions of the same patient.

In conclusion, AKI diagnostic codes recorded in the DPC data exhibited high specificity and reasonable predictive value in surgical patients treated under general anesthesia, especially in specific clinical subgroups. However, researchers should be cognizant of the low sensitivity and complement diagnosis codes with laboratory data whenever possible to minimize misclassification and improve research validity.

## SUPPLEMENTARY MATERIAL

eTable 1. Observational periods across institutions and between the DPC and SS-MIX data

eTable 2. Definition of AKI based on the KDIGO criteria

eTable 3. Validity of DPC data for identifying AKI against serum creatinine definitions using the KDIGO criteria among patients who underwent surgery (sensitivity analysis using the serum creatinine changes was limited to those occurring strictly within 7 days after surgery)

eTable 4. Validity of DPC data for identifying AKI against serum creatinine definitions using the KDIGO criteria among patients who underwent surgery (sensitivity analysis using the most current outpatient data as the baseline serum creatinine level)

eTable 5. Validity of DPC data for identifying AKI against serum creatinine definitions using the KDIGO criteria among patients who underwent surgery (sensitivity analysis using the most current inpatient data before surgery as the baseline serum creatinine level)

eTable 6. Validity of DPC data for identifying AKI against serum creatinine definitions using the KDIGO criteria among patients who underwent surgery (sensitivity analysis in which the definition of AKI was expanded to include ICD-10 N17.x, N19, and N99.0 codes recorded as in-hospital complications)

eTable 7. Literature review of AKI coding validation studies published after the introduction of the KDIGO criteria

eFigure 1. Definitions used for AKI and baseline serum creatinine in this analysis

## References

[1] Schneeweiss S, Avorn J. A review of uses of health care utilization databases for epidemiologic research on therapeutics. J Clin Epidemiol. 2005;58:323–337. 10.1016/j.jclinepi.2004.10.012 15862718

[2] Sato S, Yasunaga H. A review of studies using Japanese nationwide administrative claims databases. Ann Clin Epidemiol. 2023;5:58–64. 10.37737/ace.23008 38505730 PMC10944998

[3] van Walraven C, Austin P. Administrative database research has unique characteristics that can risk biased results. J Clin Epidemiol. 2012;65:126–131. 10.1016/j.jclinepi.2011.08.002 22075111

[4] Hoste EAJ, Kellum JA, Selby NM, . Global epidemiology and outcomes of acute kidney injury. Nat Rev Nephrol. 2018;14:607–625. 10.1038/s41581-018-0052-0 30135570

[5] Ostermann M, Lumlertgul N, Jeong R, See E, Joannidis M, James M. Acute kidney injury. Lancet. 2025;405:241–256. 10.1016/S0140-6736(24)02385-7 39826969

[6] Zarbock A, Weiss R, Albert F, . Epidemiology of surgery associated acute kidney injury (EPIS-AKI): a prospective international observational multi-center clinical study. Intensive Care Med. 2023;49:1441–1455. 10.1007/s00134-023-07169-7 37505258 PMC10709241

[7] Cheruku SR, Raphael J, Neyra JA, Fox AA. Acute kidney injury after cardiac surgery: prediction, prevention, and management. Anesthesiology. 2023;139:880–898. 10.1097/ALN.0000000000004734 37812758 PMC10841304

[8] Prowle JR, Forni LG, Bell M, . Postoperative acute kidney injury in adult non-cardiac surgery: joint consensus report of the Acute Disease Quality Initiative and PeriOperative Quality Initiative. Nat Rev Nephrol. 2021;17:605–618. 10.1038/s41581-021-00418-2 33976395 PMC8367817

[9] Scurt FG, Bose K, Mertens PR, Chatzikyrkou C, Herzog C. Cardiac surgery-associated acute kidney injury. Kidney360. 2024;5:909–926. 10.34067/KID.0000000000000466 38689404 PMC11219121

[10] Vlasschaert MEO, Bejaimal SAD, Hackam DG, . Validity of administrative database coding for kidney disease: a systematic review. Am J Kidney Dis. 2011;57:29–43. 10.1053/j.ajkd.2010.08.031 21184918

[11] Grams ME, Waikar SS, MacMahon B, Whelton S, Ballew SH, Coresh J. Performance and limitations of administrative data in the identification of AKI. Clin J Am Soc Nephrol. 2014;9:682–689. 10.2215/CJN.07650713 24458075 PMC3974361

[12] Logan R, Davey P, De Souza N, Baird D, Guthrie B, Bell S. Assessing the accuracy of ICD-10 coding for measuring rates of and mortality from acute kidney injury and the impact of electronic alerts: an observational cohort study. Clin Kidney J. 2020;13:1083–1090. 10.1093/ckj/sfz117 33391753 PMC7769533

[13] Campbell CA, Li L, Kotwal S, . Under-detection of acute kidney injury in hospitalised patients: a retrospective, multi-site, longitudinal study. Intern Med J. 2020;50:307–314. 10.1111/imj.14264 30816607

[14] Rey A, Gras-Champel V, Balcaen T, Choukroun G, Masmoudi K, Liabeuf S. Use of a hospital administrative database to identify and characterize community-acquired, hospital-acquired and drug-induced acute kidney injury. J Nephrol. 2022;35:955–968. 10.1007/s40620-021-01174-z 34618334

[15] Mitsuboshi S, Imai S, Tsuchiya M, Kizaki H, Hori S. Accuracy of diagnostic coding for acute kidney injury in Japan-analysis of a Japanese hospital-based database. Pharmacoepidemiol Drug Saf. 2025;34:e70146. 10.1002/pds.70146 40213924 PMC11987052

[16] Zhang J, Drawz PE, Zhu Y, Hultman G, Simon G, Melton GB. Validation of administrative coding and clinical notes for hospital-acquired acute kidney injury in adults. AMIA Annu Symp Proc. 2021;2021:1234–1243. 35308921 PMC8861756

[17] Molnar AO, van Walraven C, McArthur E, Fergusson D, Garg AX, Knoll G. Validation of administrative database codes for acute kidney injury in kidney transplant recipients. Can J Kidney Health Dis. 2016;3:18. 10.1186/s40697-016-0108-7 27057318 PMC4823855

[18] Kidney Disease: Improving Global Outcomes (KDIGO) CKD Work Group. KDIGO 2024 clinical practice guideline for the evaluation and management of chronic kidney disease. Kidney Int. 2024;105:S117–S314. 10.1016/j.kint.2023.10.018 38490803

[19] Yasunaga H. Updated information on the Diagnosis Procedure Combination data. Ann Clin Epidemiol. 2024;6:106–110. 10.37737/ace.24015 39726797 PMC11668689

[20] Kimura M, Nakayasu K, Ohshima Y, . SS-MIX: a ministry project to promote standardized healthcare information exchange. Methods Inf Med. 2011;50:131–139. 10.3414/ME10-01-0015 21206962

[21] Hiramatsu K, Barrett A, Miyata Y; PhRMA Japan Medical Affairs Committee Working Group 1. Current status, challenges, and future perspectives of real-world data and real-world evidence in Japan. Drugs Real World Outcomes. 2021;8:459–480. 10.1007/s40801-021-00266-3 34148219 PMC8605941

[22] Cohen JF, Korevaar DA, Altman DG, . STARD 2015 guidelines for reporting diagnostic accuracy studies: explanation and elaboration. BMJ Open. 2016;6:e012799. 10.1136/bmjopen-2016-012799 28137831 PMC5128957

[23] Siew ED, Ikizler TA, Matheny ME, . Estimating baseline kidney function in hospitalized patients with impaired kidney function. Clin J Am Soc Nephrol. 2012;7:712–719. 10.2215/CJN.10821011 22422536 PMC3338282

[24] Matsuo S, Imai E, Horio M, . Revised equations for estimated GFR from serum creatinine in Japan. Am J Kidney Dis. 2009;53:982–992. 10.1053/j.ajkd.2008.12.034 19339088

[25] Quan H, Sundararajan V, Halfon P, . Coding algorithms for defining comorbidities in ICD-9-CM and ICD-10 administrative data. Med Care. 2005;43:1130–1139. 10.1097/01.mlr.0000182534.19832.83 16224307

[26] Grimes DA, Schulz KF. Refining clinical diagnosis with likelihood ratios. Lancet. 2005;365:1500–1505. 10.1016/S0140-6736(05)66422-7 15850636

[27] Graversen HV, Jensen SK, Vestergaard SV, Heide-Jørgensen U, Christiansen CF. Defining baseline creatinine for identification of AKI in population-based laboratory databases: a Danish nationwide cohort study. Kidney360. 2022;3:232–241. 10.34067/KID.0006082021 35373126 PMC8967652

[28] Burns EM, Rigby E, Mamidanna R, . Systematic review of discharge coding accuracy. J Public Health (Oxf). 2012;34:138–148. 10.1093/pubmed/fdr054 21795302 PMC3285117

[29] Mizota T, Dong L, Takeda C, . Transient acute kidney injury after major abdominal surgery increases chronic kidney disease risk and 1-year mortality. J Crit Care. 2019;50:17–22. 10.1016/j.jcrc.2018.11.008 30469043

[30] Yamada H, Yanagita M. Global perspectives in acute kidney injury: Japan. Kidney360. 2022;3:1099–1104. 10.34067/KID.0007892021 35845320 PMC9255879

[31] Lewington AJP, Cerdá J, Mehta RL. Raising awareness of acute kidney injury: a global perspective of a silent killer. Kidney Int. 2013;84:457–467. 10.1038/ki.2013.153 23636171 PMC3758780

[32] Han L, Li H, Luo L, . Unexpectedly high rate of unrecognized acute kidney injury and its trend over the past 14 years. Sci Rep. 2025;15:6305. 10.1038/s41598-025-88732-8 39984512 PMC11845613

