# Supplementary material for: Validation of Recorded Diagnoses of Acute Kidney Injury Among Surgical Patients in the Japanese Diagnosis Procedure Combination Database
Source: J Epidemiol. 2026 Jun 5;36(6):199–204. doi: 10.2188/jea.JE20250387 (PMC13158353; doi:10.2188/jea.JE20250387)
Supplement: Supplementary file 1 [file je-36-199-s001.pdf]

eTable 1. Observational periods across institutions and between the DPC and SS-MIX data

Institute	Number of beds	Region	Data	Data availability period	Patient inclusion period
A	Approximately 1,300	Kyushu	DPC	January 2023 to December 2024	January 2023 to December 2024
			SS-MIX	April 2019 to February 2025	
B	Approximately 300	Kyushu	DPC	January 2019 to December 2024	September 2021 to December 2024
			SS-MIX	September 2021 to January 2025	
C	Approximately 300	Kanto	DPC	December 2023 to May 2024	December 2023 to May 2024
			SS-MIX	December 2023 to May 2024	

DPC, Diagnosis Procedure Combination; SS-MIX, Standardized Structured Medical Record Information Exchange.

eTable 2. Definition of AKI based on the KDIGO criteria

Stage	Serum creatinine
1	1.5–1.9 times baseline or ≥0.3 mg/dL increase
2	2.0–2.9 times baseline
3	≥3.0 times baseline<br>or increase in serum creatinine to ≥4.0 mg/dL<br>or initiation of renal replacement therapy

AKI, acute kidney injury; KDIGO, Kidney Disease: Improving Global Outcomes.

eTable 3. Validity of DPC data for identifying AKI against serum creatinine definitions using the KDIGO criteria among patients who underwent surgery (sensitivity analysis using the serum creatinine changes was limited to those occurring strictly within 7 days after surgery)

Type of analysis, subgroup	Frequency (KDIGO-defined criteria)		Frequency (ICD-10 coding)		Sensitivity (95% CI) (%)	Specificity (95% CI) (%)	PPV (95% CI) (%)	NPV (95% CI) (%)	LR+ (95% CI)	LR− (95% CI)	DOR (95% CI)
	n	(%)	n	(%)							
**Overall**	533	7.2	35	0.5	4.7 (3.1–6.9)	99.9 (99.7–99.9)	71.4 (53.7–85.4)	93.1 (92.5–93.6)	32.1 (15.5–66.5)	0.95 (0.94–0.97)	33.6 (16.3–69.3)
Stages 2 and 3	108	1.5	35	0.5	18.5 (11.7–27.1)	99.8 (99.7–99.9)	57.1 (39.4–73.7)	98.8 (98.5–99.0)	89.7 (47.2–170)	0.82 (0.75–0.89)	110 (55.0–219)
**Subgroup analyses**											
**eGFR category, mL/min/1.73 m²**											
≥60.0	302	5.6	7	0.1	1.7 (0.5–3.8)	>99.9 (99.9–>99.9)	71.4 (29.0–96.3)	94.5 (93.9–95.1)	42.5 (8.3–218)	0.98 (0.97–>0.99)	43.2 (9.6–[a])
30.0–59.9	199	11.0	23	1.3	7.5 (4.3–12.1)	99.5 (99.0–99.8)	65.2 (42.7–83.6)	89.7 (88.2–91.0)	15.1 (6.5–35.2)	0.93 (0.89–0.97)	16.3 (7.0–38.0)
15.0–29.9	32	23.4	5	3.6	15.6 (5.3–32.8)	>99.9 (96.5–>99.9)	100 (47.8–100)	79.5 (71.7–86.1)	[a]	0.84 (0.73–0.98)	[a]
**Type of admission**											
Planned	502	6.9	30	0.4	4.2 (2.6–6.3)	99.9 (99.7–99.9)	70.0 (50.6–85.3)	93.4 (92.8–93.9)	31.4 (14.5–68.3)	0.96 (0.94–0.98)	32.8 (15.2–70.6)
Emergent	31	29.0	5	4.7	12.9 (3.6–29.8)	98.7 (92.9–>99.9)	80.0 (28.4–99.5)	73.5 (63.9–81.8)	9.8 (1.1–84.3)	0.88 (0.77–>0.99)	11.1 (1.6–[a])
**Type of surgery**											
Cardiovascular	161	22.7	31	4.4	14.9 (9.8–21.4)	98.7 (97.4–99.5)	77.4 (58.9–90.4)	79.8 (76.6–82.8)	11.7 (5.1–26.6)	0.86 (0.81–0.92)	13.6 (5.9–31.4)
Non-cardiovascular	372	5.6	4	0.1	0.3 (<0.1–1.5)	>99.9 (99.9–>99.9)	25.0 (0.6–80.6)	94.4 (93.9–95.0)	5.6 (0.6–54.1)	>0.99 (0.99–>0.99)	5.7 (<0.1–39.6)

AKI, acute kidney injury; CI, confidence interval; DOR, diagnostic odds ratio; eGFR, estimated glomerular filtration rate; ICD-10, International Classification of Diseases, 10th Revision; KDIGO, Kidney Disease: Improving Global Outcomes; LR+, positive likelihood ratio; LR−, negative likelihood ratio; NPV, negative predictive value; PPV, positive predictive value.

[a] Results not shown due to infinite estimates.

eTable 4. Validity of DPC data for identifying AKI against serum creatinine definitions using the KDIGO criteria among patients who underwent surgery (sensitivity analysis using the most current outpatient data as the baseline serum creatinine level)

Type of analysis, subgroup	Frequency (KDIGO-defined criteria)		Frequency (ICD-10 coding)		Sensitivity (95% CI) (%)	Specificity (95% CI) (%)	PPV (95% CI) (%)	NPV (95% CI) (%)	LR+ (95% CI)	LR− (95% CI)	DOR (95% CI)
	n	(%)	n	(%)							
**Overall**	670	9.1	35	0.5	3.7 (2.4–5.5)	99.9 (99.7–99.9)	71.4 (53.7–85.4)	91.2 (90.5–91.8)	25.0 (12.1–51.8)	0.96 (0.95–0.98)	25.9 (12.6–53.4)
Stages 2 and 3	166	2.3	35	0.5	12.7 (8.0–18.7)	99.8 (99.7–99.9)	60.0 (42.1–76.1)	98.0 (97.7–98.3)	65.1 (33.7–126)	0.88 (0.83–0.93)	74.4 (37.5–147)
**Subgroup analyses**											
**eGFR category, mL/min/1.73 m²**											
≥60.0	410	7.5	7	0.1	1.2 (0.4–2.8)	>99.9 (99.9–>99.9)	71.4 (29.0–96.3)	92.6 (91.8–93.2)	30.7 (6.0–158)	0.99 (0.98–>0.99)	31.1 (6.9–[a])
30.0–59.9	232	13.0	21	1.2	6.9 (4.0–11.0)	99.7 (99.3–99.9)	76.2 (52.8–91.8)	87.8 (86.2–89.3)	21.5 (7.9–58.1)	0.93 (0.90–0.97)	23.0 (8.7–61.0)
15.0–29.9	28	20.3	7	5.1	14.3 (4.0–32.7)	97.3 (92.2–99.4)	57.1 (18.4–90.1)	81.7 (74.0–87.9)	5.2 (1.2–22.1)	0.88 (0.76–>0.99)	5.9 (1.4–25.4)
**Type of admission**											
Planned	636	8.8	30	0.4	3.3 (2.1–5.0)	99.9 (99.7–99.9)	70.0 (50.6–85.3)	91.5 (90.8–92.1)	24.3 (11.2–52.9)	0.97 (0.95–0.98)	25.1 (11.7–54.1)
Emergent	34	32.4	5	4.8	11.8 (3.3–27.5)	98.6 (92.4–>99.9)	80.0 (28.4–99.5)	70.0 (60.0–78.8)	8.4 (1.0–71.9)	0.90 (0.79–>0.99)	9.3 (1.3–[a])
**Type of surgery**											
Cardiovascular	167	23.6	31	4.4	13.8 (8.9–19.9)	98.5 (97.1–99.4)	74.2 (55.4–88.1)	78.8 (75.5–81.8)	9.3 (4.3–20.5)	0.88 (0.82–0.93)	10.7 (4.8–23.9)
Non-cardiovascular	503	7.5	4	0.1	0.4 (<0.1–1.4)	>99.9 (99.9–>99.9)	50.0 (6.8–93.2)	92.5 (91.8–93.1)	12.2 (1.7–86.8)	>0.99 (0.99–>0.99)	12.3 (2.2–69.9)

AKI, acute kidney injury; CI, confidence interval; DOR, diagnostic odds ratio; eGFR, estimated glomerular filtration rate; ICD-10, International Classification of Diseases, 10th Revision; KDIGO, Kidney Disease: Improving Global Outcomes; LR+, positive likelihood ratio; LR−, negative likelihood ratio; NPV, negative predictive value; PPV, positive predictive value.

[a] Results not shown due to infinite estimates.

eTable 5. Validity of DPC data for identifying AKI against serum creatinine definitions using the KDIGO criteria among patients who underwent surgery (sensitivity analysis using the most current inpatient data before surgery as the baseline serum creatinine level)

Type of analysis, subgroup	Frequency (KDIGO-defined criteria)		Frequency (ICD-10 coding)		Sensitivity (95% CI) (%)	Specificity (95% CI) (%)	PPV (95% CI) (%)	NPV (95% CI) (%)	LR+ (95% CI)	LR− (95% CI)	DOR (95% CI)
	n	(%)	n	(%)							
**Overall**	590	15.2	30	0.8	3.7 (2.4–5.6)	99.8 (99.5–99.9)	73.3 (54.1–87.7)	85.3 (84.1–86.4)	15.4 (6.9–34.4)	0.97 (0.95–0.98)	16.0 (7.2–35.3)
Stages 2 and 3	134	3.4	30	0.8	14.9 (9.4–22.1)	99.7 (99.5–99.9)	66.7 (47.2–82.7)	97.0 (96.5–97.6)	56.1 (26.8–118)	0.85 (0.80–0.92)	65.8 (30.6–141)
**Subgroup analyses**											
**eGFR category, mL/min/1.73 m²**											
≥60.0	351	12.9	5	0.2	1.1 (0.3–2.9)	>99.9 (99.8–>99.9)	80.0 (28.4–99.5)	87.2 (85.9–88.5)	27.0 (3.0–241)	0.99 (0.98–>0.99)	27.3 (4.1–[a])
30.0–59.9	204	19.3	20	1.9	6.9 (3.8–11.2)	99.3 (98.5–99.7)	70.0 (45.7–88.1)	81.7 (79.2–84.0)	9.8 (3.8–25.1)	0.94 (0.90–0.97)	10.4 (4.1–26.6)
15.0–29.9	35	31.5	5	4.5	11.4 (3.2–26.7)	98.7 (92.9–>99.9)	80.0 (28.4–99.5)	70.8 (61.1–79.2)	8.7 (1.0–74.9)	0.90 (0.80–>0.99)	9.7 (1.4–[a])
**Type of admission**											
Planned	571	14.8	28	0.7	3.5 (2.2–5.4)	99.8 (99.5–99.9)	71.4 (51.3–86.8)	85.6 (84.4–86.7)	14.3 (6.4–32.4)	0.97 (0.95–0.98)	14.8 (6.6–33.1)
Emergent	19	41.3	2	4.3	10.5 (1.3–33.1)	>99.9 (87.2–>99.9)	100 (15.8–100)	61.4 (45.5–75.6)	[a]	0.90 (0.77–>0.99)	[a]
**Type of surgery**											
Cardiovascular	161	25.6	27	4.3	11.8 (7.3–17.8)	98.3 (96.7–99.3)	70.4 (49.8–86.2)	76.5 (72.9–79.8)	6.9 (3.1–15.5)	0.90 (0.85–0.95)	7.7 (3.4–17.6)
Non-cardiovascular	429	13.1	3	0.1	0.7 (0.1–2.0)	>99.9 (99.9–>99.9)	100 (29.2–100)	86.9 (85.7–88.1)	[a]	0.99 (0.99–>0.99)	[a]

AKI, acute kidney injury; CI, confidence interval; DOR, diagnostic odds ratio; eGFR, estimated glomerular filtration rate; ICD-10, International Classification of Diseases, 10th Revision; KDIGO, Kidney Disease: Improving Global Outcomes; LR+, positive likelihood ratio; LR−, negative likelihood ratio; NPV, negative predictive value; PPV, positive predictive value.

[a] Results not shown due to infinite estimates

eTable 6. Validity of DPC data for identifying AKI against serum creatinine definitions using the KDIGO criteria among patients who underwent surgery (sensitivity analysis in which the definition of AKI was expanded to include ICD-10 N17.x, N19, and N99.0 codes recorded as in-hospital complications)

Type of analysis, subgroup	Frequency (KDIGO-defined criteria)		Frequency (ICD-10 coding)		Sensitivity (95% CI) (%)	Specificity (95% CI) (%)	PPV (95% CI) (%)	NPV (95% CI) (%)	LR+ (95% CI)	LR− (95% CI)	DOR (95% CI)
	n	(%)	n	(%)							
**Overall**	663	9.0	39	0.5	4.2 (2.8–6.1)	99.8 (99.7–99.9)	71.8 (55.1–85.0)	91.3 (90.7–92.0)	25.8 (12.9–51.5)	0.96 (0.94–0.98)	26.9 (13.5–53.5)
Stages 2 and 3	159	2.2	39	0.5	13.2 (8.4–19.5)	99.8 (99.6–99.9)	53.8 (37.2–69.9)	98.1 (97.8–98.4)	52.9 (28.8–97.4)	0.87 (0.82–0.92)	60.8 (32.0–116)
**Subgroup analyses**											
**eGFR category, mL/min/1.73 m²**											
≥60.0	382	7.0	8	0.1	1.3 (0.4–3.0)	99.9 (99.8–>99.9)	62.5 (24.5–91.5)	93.1 (92.3–93.7)	22.0 (5.3–91.9)	0.99 (0.98–>0.99)	22.3 (5.9–84.8)
30.0–59.9	245	13.6	26	1.4	7.4 (4.4–11.4)	99.5 (99.0–99.8)	69.2 (48.2–85.7)	87.2 (85.6–88.7)	14.3 (6.3–32.6)	0.93 (0.90–0.97)	15.4 (6.8–35.0)
15.0–29.9	36	26.3	5	3.6	13.9 (4.7–29.5)	>99.9 (96.4–>99.9)	100 (47.8–100)	76.5 (68.4–83.5)	[a]	0.86 (0.76–0.98)	[a]
**Type of admission**											
Planned	630	8.7	33	0.5	3.5 (2.2–5.2)	99.8 (99.7–99.9)	66.7 (48.2–82.0)	91.6 (90.9–92.2)	21.1 (10.3–43.2)	0.97 (0.95–0.98)	21.8 (10.7–44.5)
Emergent	33	30.8	6	5.6	18.2 (7.0–35.5)	>99.9 (95.1–>99.9)	100 (54.1–100)	73.3 (63.5–81.6)	[a]	0.82 (0.70–0.96)	[a]
**Type of surgery**											
Cardiovascular	172	24.2	34	4.8	14.0 (9.2–20.0)	98.1 (96.6–99.1)	70.6 (52.5–84.9)	78.1 (74.8–81.2)	7.5 (3.7–15.4)	0.88 (0.83–0.93)	8.6 (4.1–18.0)
Non-cardiovascular	491	7.4	5	0.1	0.8 (0.2–2.1)	>99.9 (99.9–>99.9)	80.0 (28.4–99.5)	92.7 (92.0–93.3)	50.3 (5.6–449)	0.99 (0.98–>0.99)	50.7 (7.6–[a])

AKI, acute kidney injury; CI, confidence interval; DOR, diagnostic odds ratio; eGFR, estimated glomerular filtration rate; ICD-10, International Classification of Diseases, 10th Revision; KDIGO, Kidney Disease: Improving Global Outcomes; LR+, positive likelihood ratio; LR−, negative likelihood ratio; NPV, negative predictive value; PPV, positive predictive value.

[a] Results not shown due to infinite estimates

eTable 7. Literature review of AKI coding validation studies published after the introduction of the KDIGO criteria

Study (author, year [ref]-subgroup)	Validation period	Country of origin	Study sample	Administrative codes used to identify AKI	Reference standard for AKI diagnosis	Definition of baseline serum creatinine	Sensitivity (%)	Specificity (%)	PPV (%)	NPV (%)
Mitsuboshi et al. 2025 [17]	2014–2022	Japan	All admissions during the study period	ICD-10: N17, N14	KDIGO criteria (EMR review, SCr only)	Two SCr values within 7 days; earlier used as baseline	0.3	>99	12	85
Grams et al. 2014 [11]-1	2002–2008	United States	Participants with Atherosclerosis Risk in Communities study	ICD-9-CM: 584.x, V45.1, V56, 39.95, 54.98 or ICD-10: N17.x, Z99.2, Z49, Z45.2	KDIGO criteria (EMR review, SCr and urine output)	Average outpatient SCr measured 10–365 days before index admission	12	99	84	69
Grams et al. 2014 [11]-2	1996–2008	United States	Participants with Atherosclerosis Risk in Communities study	ICD-9-CM: 584.x, V45.1, V56, 39.95, 54.98 or ICD-10: N17.x, Z99.2, Z49, Z45.2	KDIGO criteria (Chart review, SCr only)	Average outpatient SCr measured 10–365 days before index admission	17	>99	92	82
Grams et al. 2014 [11]-3	2002–2008	United States	Participants with Atherosclerosis Risk in Communities study	ICD-9-CM: 584.x, V45.1, V56, 39.95, 54.98 or ICD-10: N17.x, Z99.2, Z49, Z45.2	KDIGO criteria (EMR review, SCr only)	Average outpatient SCr measured 10–365 days before index admission	17	99	72	84

Campbell et al. 2019 [15]-1	2009–2013	Australia	All admissions in 2009	ICD-10-AM: N00, N10, N17, E10.29, E11.29, E13.29, E14.29, O90.4, O08.4, N99.0	NHS England algorithm (EMR review, SCr only)	Median SCr measured 8–365 days prior; if unavailable, lowest SCr during 0–7 or 0–2 days prior	18	NA	NA	NA
Logan et al. 2020 [14]-1	2013–2017	United Kingdom	All admissions during the study period	ICD-10: N17.x	KDIGO criteria (EMR review, SCr only)	Median SCr measured 8–365 days prior; if unavailable, lowest SCr in 0–7 or 0–2 days prior	26	99	76	93
Molnar et al. 2016 [13]-1	2003–2012	Canada	Kidney transplant recipients	ICD-10: N17.x as all diagnosis	Acute Kidney Injury Network staging system (EMR review, SCr only)	SCr between 2 weeks and 6 months before index admission	28	97	89	62
Campbell et al. 2019 [15]-2	2009–2013	Australia	All admissions in 2013	ICD-10-AM: N00, N10, N17, E10.29, E11.29, E13.29, E14.29, O90.4, O08.4, N99.0	NHS England algorithm (EMR review, SCr only)	Median SCr 8–365 days prior; if unavailable, lowest SCr during 0–7 or 0–2 days prior	30	NA	NA	NA

Campbell et al. 2019 [15]-3	2009–2013	Australia	All admissions in 2009	ICD-10-AM: N00, N10, N17, E10.29, E11.29, E13.29, E14.29, O90.4, O08.4, N99.0	NHS England algorithm (EMR review, SCr only, stage 2 or greater)	Median SCr measured 8–365 days prior; if unavailable, lowest SCr in 0–7 or 0–2 days prior	35	NA	NA	NA
Molnar et al. 2016 [13]-2	2003–2012	Canada	Kidney transplant recipients	ICD-10: N17.x as all diagnosis	Acute Kidney Injury Network staging system (EMR review, SCr only, stage 2 or greater)	SCr measured between 2 weeks and 6 months before index admission	42	91	43	90
Logan et al. 2020 [14]-2	2013–2017	United Kingdom	All admissions during the study period	ICD-10: N17.x	KDIGO criteria (EMR review, SCr only, stage 2 or greater)	Median SCr 8–365 days prior; if unavailable, lowest SCr in 0–7 or 0–2 days prior	44	98	46	98
Zhang et al. 2021 [12]	2008–2019	United States	All admissions during the study period	ICD-9-CM: 584.x, V45.1, V56, 399.95, 54.98 or ICD-10: N17.x, Z49, Z45.2, Z99.2	KDIGO criteria (EMR review, SCr only)	Median and most recent SCr within 1 year before admission	49	95	51	94

Campbell et al. 2019 [15]-4	2009–2013	Australia	All admissions in 2013	ICD-10-AM: N00, N10, N17, E10.29, E11.29, E13.29, E14.29, O90.4, O08.4, N99.0	NHS England algorithm (EMR review, SCr only, stage 2 or greater)	Median SCr measured 8–365 days prior; if unavailable, lowest SCr in 0–7 or 0–2 days prior	53	NA	NA	NA
Rey et al. 2022 [16]	2019	France	All admissions during the study period	ICD-10: I12.0, I13.9, K76.7, N05.5, N05.6, N05.9, N13.9, N17.0, N17.1, N17.2, N17.8, N17.9, N19, N99.0, P96.0, R39.2, T79.5, N14.1, N14.2, N14.4	KDIGO criteria (EMR review, SCr only)	Most recent serum creatinine before index admission	57	95	95	57

AKI, acute kidney injury; CABG, coronary bypass graft; EMR, electronic medical record; ICD, International Classification of Diseases; KDIGO, Kidney Disease: Improving Global Outcomes; NA, not applicable; NHS, national health service; NPV, negative predictive value; PPV, positive predictive value; SCr, serum creatinine.

e Figure 1. Definitions used for AKI and baseline serum creatinine in this analysis

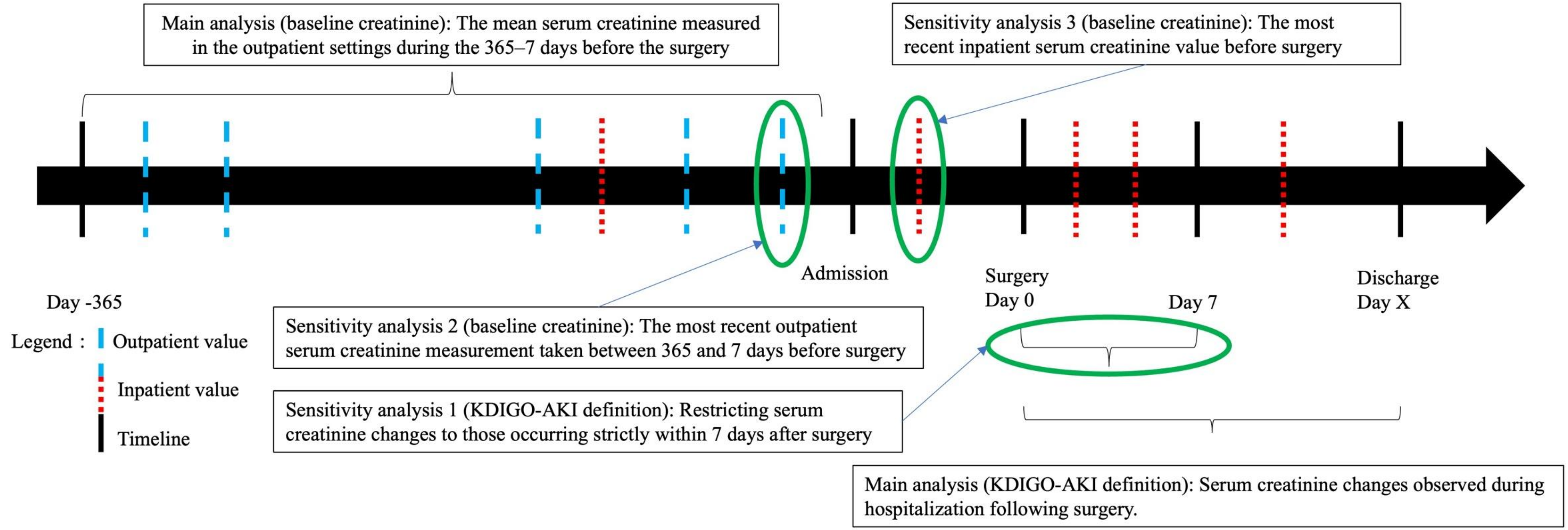

This diagram illustrates the time windows and selection rationale for serum creatinine values in patients undergoing surgery. The blue dashed bars represent outpatient serum creatinine tests, and the red dotted bars represent inpatient serum creatinine tests. The main analysis incorporated the mean outpatient serum creatinine values obtained between 365 and 7 days prior to surgery as the baseline, with follow-up serum creatinine values assessed during hospitalization after surgery. Sensitivity analysis 1 restricted follow-up serum creatinine to within 7 days after surgery (changing the KDIGO-AKI definition). Sensitivity analysis 2 used the most recent outpatient serum creatinine value from the baseline window (changing the baseline creatinine value). Sensitivity analysis 3 used the most recent inpatient serum creatinine value before surgery (changing the baseline creatinine value). AKI, acute kidney injury; KDIGO, Kidney Disease: Improving Global Outcomes.
